# PCSK9 Regulates Nox2-Mediated Platelet Activation via CD36 Receptor in Patients with Atrial Fibrillation

**DOI:** 10.3390/antiox9040296

**Published:** 2020-04-02

**Authors:** Vittoria Cammisotto, Daniele Pastori, Cristina Nocella, Simona Bartimoccia, Valentina Castellani, Cinzia Marchese, Antonio Sili Scavalli, Evaristo Ettorre, Nicola Viceconte, Francesco Violi, Pasquale Pignatelli, Roberto Carnevale

**Affiliations:** 1 Department of General Surgery and Surgical Speciality Paride Stefanini, Sapienza University of Rome, 00161 Rome, Italy; vittoria.cammisotto@uniroma1.it; 2 Department of Clinical, Internal Medicine and Cardiovascular Sciences, Sapienza University of Rome, Viale del Policlinico 155, 00161 Rome, Italy; daniele.pastori@uniroma1.it (D.P.); cristina.nocella@uniroma1.it (C.N.); simona.bartimoccia@uniroma1.it (S.B.); valentina.castellani@virgilio.it (V.C.); antonio.siliscavalli@uniroma1.it (A.S.S.); evaristo.ettorre@uniroma1.it (E.E.); nicola.viceconte@uniroma1.it (N.V.); francesco.violi@uniroma1.it (F.V.); 3 Department of Experimental Medicine, Sapienza University of Rome, 00161 Rome, Italy; cinzia.marchese@uniroma1.it; 4 Mediterranea Cardiocentro, 80122 Naples, Italy; roberto.carnevale@uniroma1.it; 5 Department of Medical-Surgical Sciences and Biotechnologies, Sapienza University of Rome, 04100 Latina, Italy

**Keywords:** PCSK9, ROS, oxidative stress, platelets, CD36

## Abstract

Background: High levels of proprotein convertase subtilisin/kexin 9 (PCSK9) is predictive of cardiovascular events (CVEs) in atrial fibrillation (AF). We hypothesized that PCSK9 may directly induce platelet activation (PA). Methods: We measured platelet aggregation, recruitment, Thromboxane B2 (TxB2) formation and soluble P-selectin levels as markers of PA and soluble Nox2-derived peptide (sNox2-dp), H_2_O_2_, isoprostanes and oxidized Low-Density-Lipoprotein (oxLDL) to analyze oxidative stress (OS) in 88 patients having PCSK9 values < (*n* = 44) or > (*n* = 44) 1.2 ng/mL, balanced for age, sex and cardiovascular risk factors. Furthermore, we investigated if normal (*n* = 5) platelets incubated with PCSK9 (1.0–2.0 ng/mL) alone or with LDL (50 µg/mL) displayed changes of PA, OS and down-stream signaling. Results: PA and OS markers were significantly higher in patients with PCSK9 levels > 1.2 ng/mL compared to those with values < 1.2 ng/mL (*p* < 0.001). Levels of PCSK9 significantly correlated with markers of PA and OS. Platelets incubation with PCSK9 increased PA, OS and p38, p47 and Phospholipase A2 (PLA2) phosphorylation. These changes were amplified by adding LDL and blunted by CD36 or Nox2 inhibitors. Co-immunoprecipitation analysis revealed an immune complex of PCSK9 with CD36. Conclusions: We provide the first evidence that PCSK9, at concentration found in the circulation of AF patients, directly interacts with platelets via CD36 receptor and activating Nox2: this effect is amplified in presence of LDL.

## 1. Introduction

Proprotein convertase subtilisin/kexin type 9 (PCSK9) is principally present in the liver, where it is able to modulate Low-Density-Lipoprotein (LDL) cholesterol uptake acting on its LDL receptor (LDL-R). The gain of function variants of PCSK9 gene is associated with increased levels of circulating LDL cholesterol, and a higher risk of cardiovascular events (CVE) [1]. Recent evidence showed that PCSK9 possesses pro-atherogenic functions independently from its regulatory effect on plasma lipid levels. Thus, PCSK9 is highly expressed in vascular smooth muscle cells, in human atherosclerotic plaques and its expression is regulated by several pro-atherogenic mediators, such as tumor necrosis factor α (TNF-α), reactive oxygen species (ROS) via NADPH oxidase activation, and damaged mitochondrial DNA [2,3].

A recent prospective study investigated PCSK9 levels in atrial fibrillation (AF) patients and revealed that high plasma levels of PCSK9 were significantly associated with urinary 11-dehydro-Thromboxane B2 (11-dh-TxB2), a marker of systemic COX1 activation, suggesting an interplay between PCSK9 and platelet activation [4]. Accordingly, PCSK9 was associated with increased platelet aggregation in patients with acute coronary syndrome [5] and treatment with anti-PCSK9 mAb was found to modulate platelet reactivity [6]. The direct involvement of PCSK9 in platelet activation (PA) was confirmed by PCSK9-/- animal models of mice that presented reduced FeCl3 injury-induced carotid artery thrombosis [7,8]. Furthermore, human recombinant PCSK9 (hrPCSK9), significantly enhanced PA but the mechanism was not fully explored [8]. Among the mechanisms involved in PA, ROS and eventually oxidized LDL (ox-LDL) play an important role via triggering the intra-cellular signaling of platelet activation [9]. As inhibition of PCSK9 is associated with impaired production of ox-LDL, it would be possible to postulate that ox-LDL down-regulation might be responsible for PCSK9-induced PA [10].

Based on this hypothesis, aim of the study was to evaluate if PCSK9, in the range of concentration achievable in human circulation, may influence PA.

In particular, to explore this issue, we measured bio-markers of PA and OS in AF patients with PCSK9 values < or > to 1.2 ng/mL. Furthermore, to substantiate that PCSK9 could be biologically active on platelet activation, we performed in vitro studies to assess (i) the interplay between PCSK9 and platelet activation, and (ii) oxidative stress, and (iii) to investigate the molecular mechanism involved.

## 2. Materials and Methods

We performed a cross-sectional study including 88 out of 1135 non-valvular AF patients included in the ATHERO-AF cohort. Patients were balanced for sex, age and cardiovascular risk factors and clinical characteristics are reported in Table 1.

Patients were recruited from the Athero-thrombosis Centre of the Department of Clinical, Internal Medicine, Anesthesiology and Cardiovascular Sciences, Sapienza-University of Rome, for monitoring and management of antithrombotic therapies. All patients were treated with vitamin K antagonists and none of patients were on treatment with antiplatelet drugs. All patients provided written informed consent at baseline. Ex vivo platelet aggregation and recruitment were performed using citrated blood samples, which were taken and treated as above reported while for marker of platelet activation and oxidative stress analysis, the blood was collected in tube without anticoagulant and centrifuged at 300 g for 10 min at room temperature (RT).

The study protocol was approved by the local ethical board of Sapienza-University of Rome (ethical protocol code is 1306/2007) and was conducted according to principles of the Declaration of Helsinki.

### 2.1. Platelet Preparation

Blood samples adding with citrated (3.8%, 1/10 (*v: v*) were taken between 8 and 9 am from healthy subjects (HS), (*n* = 5, males 3, females 2, age 38.8 ± 7.0 years) in fasting conditions. After all subjects gave written informed consent, they were included in the study. The study protocol was approved by the local ethical board of Sapienza University of Rome and was conducted according to the principles of the Declaration of Helsinki.

Blood was centrifuged for 15 min at 180 g at RT and the supernatant obtained was separated (2 × 10^5^ platelets/μL) and represents platelet-rich plasma (PRP). To exclude leukocyte contamination, only the top 75% of the PRP was picked. Washed platelets (wPLT) were isolated from PRP by consecutive centrifugation steps (10 min at 300× *g* at RT) and resuspended in Tyrode’s buffer (137 mM NaCl, 2.7 mM KCl, 1.0 mM MgCl_2_, 1.8 mM CaCl_2_, 20 mM HEPES, 0.35% *w/v* bovine serum albumin (BSA), and 5.6 mM glucose, pH 7.35; Sigma Aldrich, St. Louis MO, USA). To prevent platelet activation, we added prostaglandin E1 (PGE1,1 µM).

### 2.2. Platelet Aggregation and Recruitment

For ex vivo study, platelet aggregation was induced with collagen (2 μg/mL, Mascia Brunelli, Italy, EU) and was measured for 8 min, subsequently platelet recruitment (PR), that is an vitro method to evaluate thrombus formation, was performed as previously described [11]. Briefly, after 8 min from the aggregation induction with collagen, an equal quantity of untreated PRP was added to each sample that increased the density of the solution; this event produced a reduction in light transmission. Therefore, the aggregation of the freshly added platelet portion in the presence of an existing aggregate was measured for 5 min and expressed as a percentage of the aggregation (percentage of light transmission) that had initially been reached [11]. Instead, for in vitro study, PRP was stimulated with a subthreshold concentration (STC, 0.25 μg/mL), the concentration of agonists was defined as the highest concentration that elicited < 20% platelet aggregation of collagen as a primer. Before activation, samples were pre-incubated (20 min at 37 °C) with Nox2 inhibitor, that specifically inhibits interactions between Nox2 and p47^phox^ (Nox2ds-tat, 10 μM; Anaspec, Fremont, CA, USA); or anti-PCSK9 (5 μM; Abcam, Cambridge, UK); anti-CD36 and anti-LOX1 (5 μM; Cayman, Ann Arbor, MI, USA; Abcam, Cambridge, UK, respectively), that are two receptors on the platelet surface that binds and internalizes oxLDL; or an inhibitor of cytosolic phospholipase A_2_ (AACOCF_3_, 1 µM; Tocris, Bristol, UK); or p38 inhibitor (SB202190, 5 µmol/L; Santa Cruz Biotechnology, Dallas, TX, USA). After incubation, platelets were treated with PCSK, in the range of concentration achievable in circulation of AF patients, (1–2 ng/mL; Selleckchem S302, Houston, TX, USA) for 5 min before of activation. Finally, samples were centrifuged for 3 min at 3000 rpm and supernatants and pellets were stored at –80 °C for analysis of H_2_O_2_, sNox2-dp, isoprostanes, TXB_2,_ ox-LDL production and p38, p47 and PLA_2_ phosphorylation, as reported below. PA was performed on PRP with Platelet Aggregation Profile–8E (Bio/Data Horsham, PA, USA), using siliconized glass cuvettes in constant stirring condition, using Born method [12]. Furthermore, to assess if PCSK9 amplified platelet activation by ox-LDL pathway we performed platelet aggregation in wPLT incubated with and without LDL (50 μg/mL). To evaluate PA in wPLT we added, immediately before to induce aggregation, CaCl_2_ and fibrinogen (1 mM and 100 μg/mL, respectively; Sigma Aldrich, St. Louis, MO, USA). Finally, all samples were centrifuged for 3 min at 3000 rpm and supernatants and pellets were separated and stored at –80 °C for analysis of sNox2-dp, H_2_O_2_, isoprostanes, TXB_2,_ ox-LDL, conjugated dienes and p38, p47 and PLA_2_ phosphorylation, as reported below.

### 2.3. Serum and Platelet sNox2-dp

Serum and platelet Nox2 were measured as a soluble Nox2-derived peptide (sNox2-dp) with an ELISA method as previously reported [13]. Briefly, the peptide is recognized by binding to a specific monoclonal antibody against the amino acid sequence (224–268) that corresponds to the extracellular membrane part of Nox2 (catalytic core of NADPH oxidase), which was released following platelet activation. The enzyme activity is measured spectrophotometrically by the increased absorbance at 450 nm. Values were expressed as pg/mL; intra-assay and inter-assay coefficients of variation were 8.95% and 9.01%, respectively.

### 2.4. Serum and Platelet H_2_O_2_ Production

The Hydrogen Peroxide (H_2_O_2_) was measured by using a colorimetric assay as described previously [14]. A standard curve of H_2_O_2_ (0–200 μM) was performed for each assay. In brief, samples were mixed with colorless colorimetric substrate (50 μL of 3,3′,5,5′-tetramethylbenzidine in 0.42 mol/L citrate buffer, pH 3.8) and and the reaction initiated by addition of horseradish peroxidase (HRP, 52.5 U/mL). After, the samples were incubated at room temperature for 20 min, and the reaction was stopped adding an acid solution (18 N sulphuric acid). The product was read at 450 nm and expressed as μM. Intra-assay and inter-assay coefficients of variation were both < 10%.

### 2.5. Platelet and Urinary 8-Iso-PGF_2α_ Assay

Platelet and urinary isoprostanes (8-iso-PGF2α) were measured by the enzyme immunoassay technology (DRG Inter-national, Springfield, NJ, USA) the values were expressed in pmol/L and pg/mg creatinine, respectively. Intra-assay and inter-assay coefficients of variation were 5.8% and 5.0%, respectively.

### 2.6. Serum Detection of Oxidization of Low-Density Lipoprotein (ox-LDL)

ox-LDL were measured by commercially available immunoassays (Cusabio, Houston, TX, USA). The optical density of samples was read at 450 nm and were expressed in mU/L. Intra-assay and inter-assay coefficients of variation were < 8% and < 10%, respectively.

### 2.7. Serum and Platelet TxB_2_ Assay

Serum and platelet TxA_2_ were analyzed by estimating its stable metabolite named TxB_2_ in the supernatant by an ELISA commercial kit (Cusabio, Houston, TX, USA), according to manufacturer instructions. The values were expressed as pg/mL × 10^8^ cells and pg/mL, respectively. Intra- and inter-assay coefficients of variation for TxB_2_ were < 8% and < 10%, respectively.

### 2.8. Serum and Platelet sP-selectin Assay

Serum and platelet sP-selectin concentrations were evaluated by a commercial immunoassay kit (Diaclone), and values were expressed as ng/mL; intra- and inter- assay coefficients of variation were 5.6% and 7.5%, respectively.

### 2.9. LDL Isolation and Determination of Conjugated Dienes

Human LDL was obtained from healthy subjects and blood was collected into tubes with 7.2 mg EDTA. Briefly, (1) blood was centrifuged at 1500× *g* for 10 min at 4 °C, (2) 250 μL of PBS adding 0.25 mM EDTA was used to stratified 750 μL of plasma, (3) tubes were centrifuged at 100,000 rpm for 7 min, (4) the upper 250 μL was removed to eliminate chylomicrons and (5) 250 μL of PBS with 0.25 mM EDTA was added again; (6) samples were centrifuged at 100,000 rpm for 2.5 h; (7) next, 250 μL of the upper layer was eliminated and (8) 150 μL of potassium bromide (KBr) (50%, *w/v*) was added, the final density obtained is 1.063 g/mL. Samples were centrifuged at 100,000 rpm for 5 h and and the fraction containing LDL was recovered. Finally, 200 μL of the fraction of LDL was dialyzed with PBS containing EDTA.

LDLs were oxidized by copper sulfate (CuSO_4_) and the degree of oxidation was evaluated by conjugated dienes formation using a UV/VIS spectrometer (PerkinElmer, Waltham, MA, USA). Measurement of the 234 nm absorption was read in the cell supernatant after oxidation and expressed as μg/mL of conjugated dienes [15].

### 2.10. Co-Immunoprecipitation Assays and Western Blot Analysis

For co-immunoprecipitation (IP), platelet pellets were suspended in RIPA buffer with protease and phosphatase inhibitors cocktail (10 μg/mL; Thermo Fisher Scientific, MA, USA) and sonicated three times (for 10 s and 70% amplitude). Once the samples were sonicated, they were incubated on ice for 30 min. After this, the samples were centrifuged at 10,000× *g* for 20 min to remove pellet residues (residues may contain unlysed cells, nuclei or unlysed organelles) and the supernatants were collected. The protein concentration of each lysate was determined by Bradford assay.

Cellular proteins (500 µg) were diluted in RIPA buffer and incubated with corresponding mouse monoclonal antibodies anti-CD36 or anti-LOX-1 (1 µg/mg protein) overnight at 4 °C. Then protein-A/G agarose (Santa Cruz Biotechnology, Dallas, TX, USA) was added to the mixtures and adequately resuspended. After incubation for 60 min at 4 °C, the mixtures were centrifuged at 2500 rpm for 5 min and pellet washed with RIPA buffer 3 times. Finally, the immunoprecipitates were dissolved in a mix solution consisting of (lysis buffer with 4X Leammli sample buffer and 20% of 2-mercaptoethanol) (Bio-Rad, Hercules, CA, USA) and heated at 100 °C for 5 min. Proteins were resolved by SDS-PAGE on 7% polyacrylamide gel and transferred on nitrocellulose membranes. After blocking with BSA (5%), membranes were incubated with anti-PCSK9, (Santa Cruz Biotechnology, Dallas, TX, USA) and incubated overnight at 4 °C.

To analyse p38, p47phox and PLA2 phosphorylation we used the same procedure but immediately after cell lysis, 30 μg/lane were separated by SDS-PAGE on 10–12% polyacrylamide gel. Moreover, membranes were incubated overnight at 4 °C with rabbit polyclonal anti-p-p47phox (1:1000; ab-795, Abcam, Cambridge, UK), anti-p-p38 and anti-p-cPLA2 antibody (1:1000; sc-7973 and sc34392, Santa Cruz Biotechnology, Dallas, TX, USA; respectively), and anti-p47phox, anti-p38 and anti-cPLA2 antibody (1:1000; sc-17845, sc-7973 and sc-376618, Santa Cruz Biotechnology, TX, USA; respectively). Then, the membranes were incubated for 1 h with HRP-conjugated secondary antibody (1:3000; Bio-Rad, Hercules, CA, USA) and the co-immune and immune complexes were detected by enhanced chemiluminescence substrate (ECL Substrates, Bio-Rad, Hercules, CA, USA). Densitometric analysis of the bands was performed using Image J software v6.0.1 (BioRad, Hercules, CA, USA). The results were expressed as arbitrary units (A.U.) and represent the mean of three independent experiments.

### 2.11. Statistical Analysis

For analysis, all data were expressed as means ± SEM. Statistical comparisons were performed using one-way ANOVA or Student *t* test and *p* values < 0.05 were considered to be statistically significant. All tests were performed using GraphPad Software-Prism7 (GraphPad Company, San Diego, CA, USA).

## 3. Results

### 3.1. Ex-Vivo Study

Clinical characteristics of AF patients enrolled for the study are summarized in Table 1.

**Figure 1:**
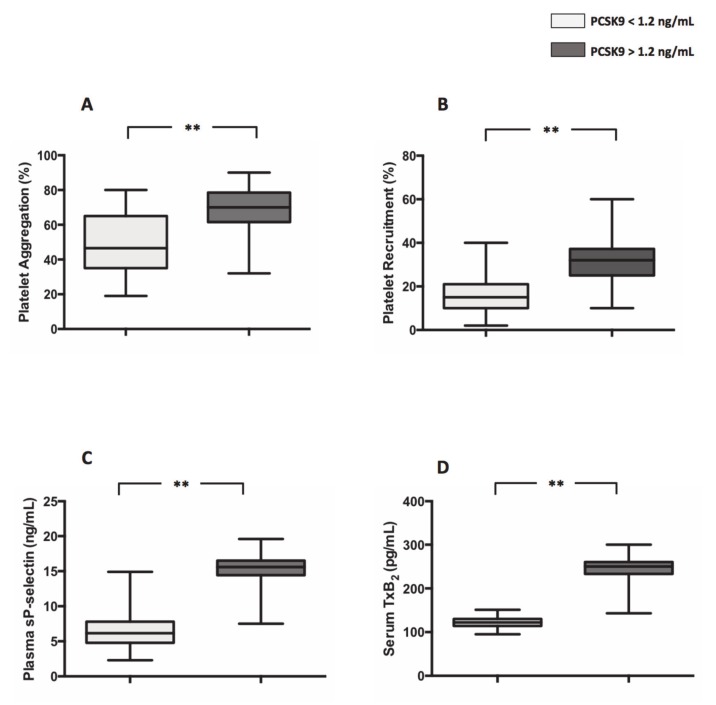
Proprotein convertase subtilisin/kexin 9PCSK9 and platelet activation in atrial fibrillation (AF) patients. Platelet aggregation (**A**), platelet recruitment (**B**), plasma soluble P-selectin (sP-selectin) (**C**) and serum TxB_2_ (**D**) in 44 AF patients with plasma levels of PCSK9 < 1.2 ng/mL and 44 AF patients with plasma levels of PCSK9 > 1.2 ng/mL. (Data are represented as median and IQR. ** *p* < 0.001).

Median values of PCSK9 in the entire population was 1.2 ng/mL, 44 having above and 44 below 1.2 ng/mL levels. Compared to patients with PCSK9 < 1.2 ng/mL, patients with plasma levels of PCSK9 > 1.2 ng/mL, displayed higher platelet aggregation, (49.8 ± 2.5% vs. 68.6 ± 1.9%; **p < 0.001) (Figure 1A), platelet recruitment (16.46 ± 1.568% vs. 31.20 ± 1.861%; **p < 0.001) (Figure 1B), sP-selectin (7.7 ± 0.6 ng/mL vs. 14.7 ± 0.45 ng/mL; **p < 0.001) (Figure 1C) and serum levels of TxB_2_ (121.5 ± 1.9 pg/mL vs. 245.5 ± 4.6 pg/mL; **p < 0.001) (Figure 1D).

**Figure 2:**
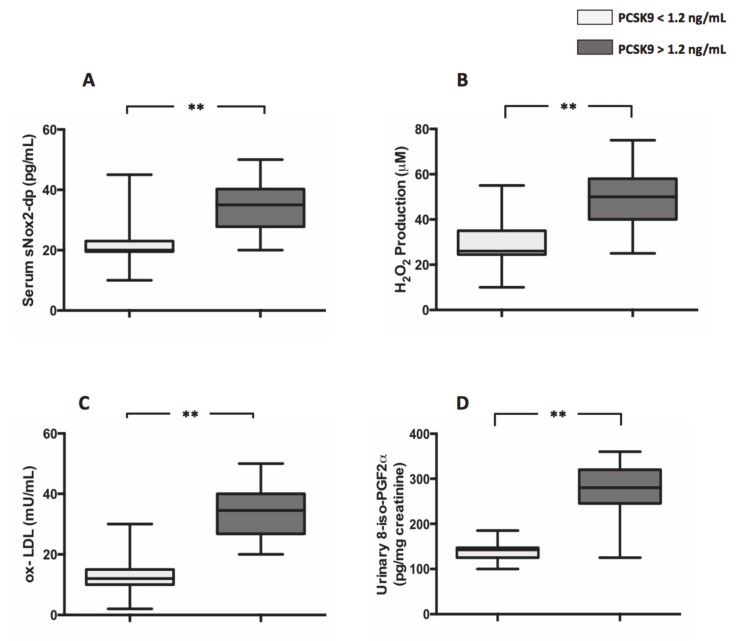
PCSK9 and oxidative stress in AF patients. Nox2 activation (**A**), H2O2 production (**B**), ox-LDL formation (**C**) and 8-iso-PGF2α (**D**) biosynthesis in 44 AF patients with plasma levels of PCSK9 < 1.2 ng/mL and 44 AF patients with plasma levels of PCSK9 > 1.2 ng/mL. (Data are represented as median and IQR. **p < 0.001).

Moreover, compared to patients with PCSK9 < 1.2 ng/mL, patients with PCSK9 >1.2 ng/mL displayed an increased levels of markers of oxidative stress such as s-Nox2dp (21.4 ± 0.9 pg/mL vs. 34.4 ± 1,1 pg/mL; **p < 0.0001) (Figure 2A), H_2_O_2_ (29.5 ± 1.3 µM vs. 48.8 ± 1.6 µM; **p < 0.001) (Figure 2B), ox-LDL (12.0 ± 0.9 mU/mL vs. 33.5 ± 1.1 mU/mL; **p < 0.001) (Figure 2C) and 8-iso-PGF2α (137.2 ± 22.0 pg/mg creatinine vs. 272.0 ± 8.6 pg/mg creatinine; **p < 0.001) (Figure 2D), respectively.

Circulating levels of PCSK9 are significantly positively associated with markers of platelet activation and oxidative stress (Table 2).

### 3.2. In Vitro Study

#### 3.2.1. PCSK9 and Platelet Activation

**Figure 3:**
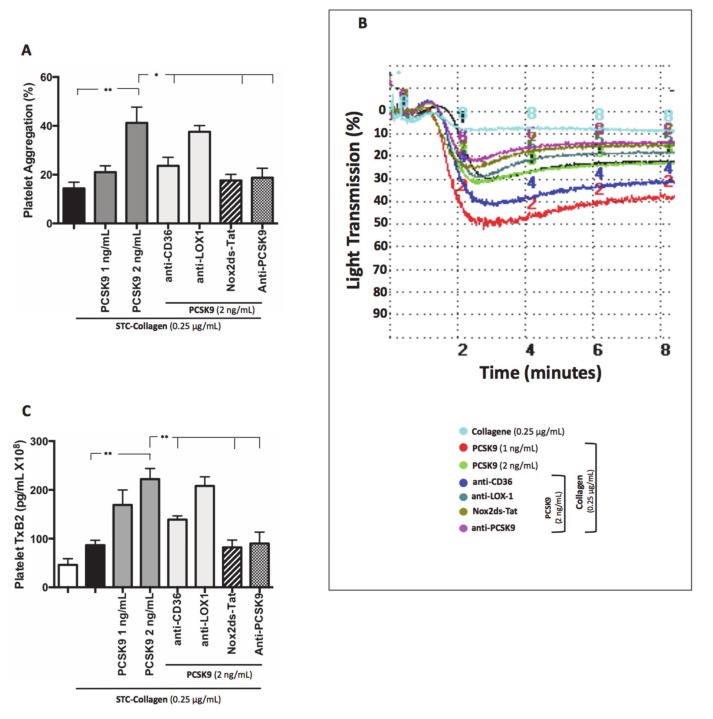
PCSK9 and platelet activation. Platelet aggregation (**A**) with relative representative tracing (**B**) and platelet TxB2 (**C**) evaluated in platelet rich plasma (PRP) treated with PCSK9 (1 and 2 ng/mL) and stimulated with subthreshold concentration (STC) of collagen (0.25 μg/mL) in presence or not of anti-CD36, anti-LOX1, Nox2ds-tat and anti-PCSK9. (*n* = 5) (*p < 0.05, **p < 0.001 for paired analyses).

Platelets co-incubated with PCSK9 and stimulated with STC collagen showed a dose-dependent increase of platelet aggregation and TxB_2_ biosynthesis compared to STC collagen platelets alone (Figure 3A–C). Such an effect was inhibited by the addition of anti-CD36, Nox2ds-tat and anti-PCSK9 (Figure 3A–C). No significant changes were observed with anti-LOX1 (Figure 3A–C). Finally, platelet incubation with inhibitors alone, without PCSK9 did not show significant variations, neither in platelet aggregation nor in TxB_2_ biosynthesis (Figure S1A–C).

#### 3.2.2. PCSK9 and Oxidative Stress

**Figure 4:**
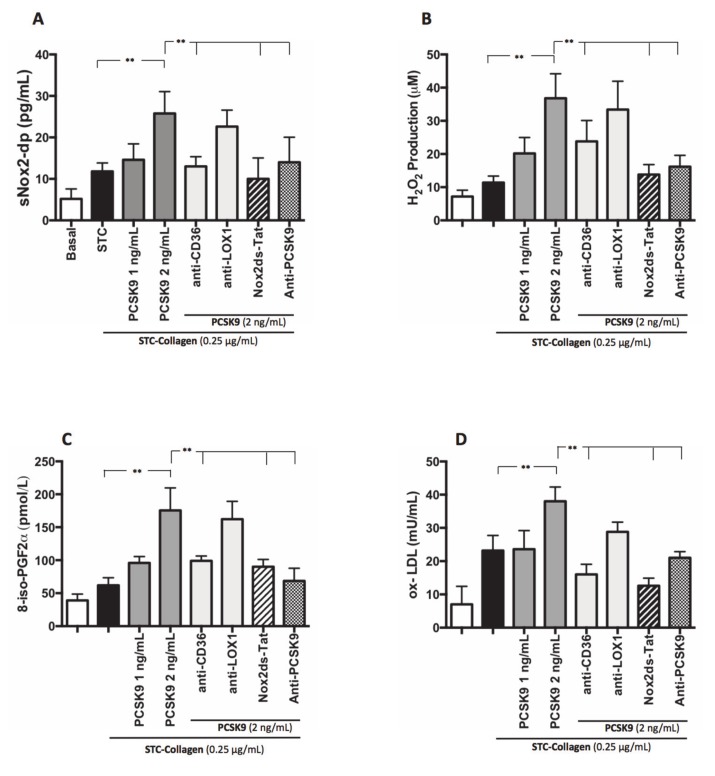
PCSK9 and oxidative stress. Nox2 activation (**A**), H_2_O_2_ production (**B**), 8-iso-PGF2α-III (**C**) and ox-LDL formation (**D**) evaluated in PRP treated with PCSK9 (1 and 2 ng/mL) and stimulated with STC of collagen (0.25 μg/mL) in presence or less of anti-CD36, anti-LOX1, Nox2ds-tat and anti-PCSK9. (*n* = 5) (**p < 0.001 for paired analyses).

Similarly, platelets co-incubated with PCSK9 and stimulated with STC collagen showed a dose-dependent increase of Nox2 activation, H_2_O_2_ production, isoprostanes biosynthesis and ox-LDL formation compared to STC collagen platelets alone (Figure 4A–D). Such effect was inhibited by anti-CD36, Nox2ds-tat and anti-PCSK9 (Figure 4A,B). No significant changes were observed with anti-LOX1 (Figure 4A,B).

#### 3.2.3. Intra-Signalling Pathway of Platelet Activation PCSK9-Mediated p38MAP Kinase, p47^phox^ and cPLA_2_ Phosphorylation

**Figure 5:**
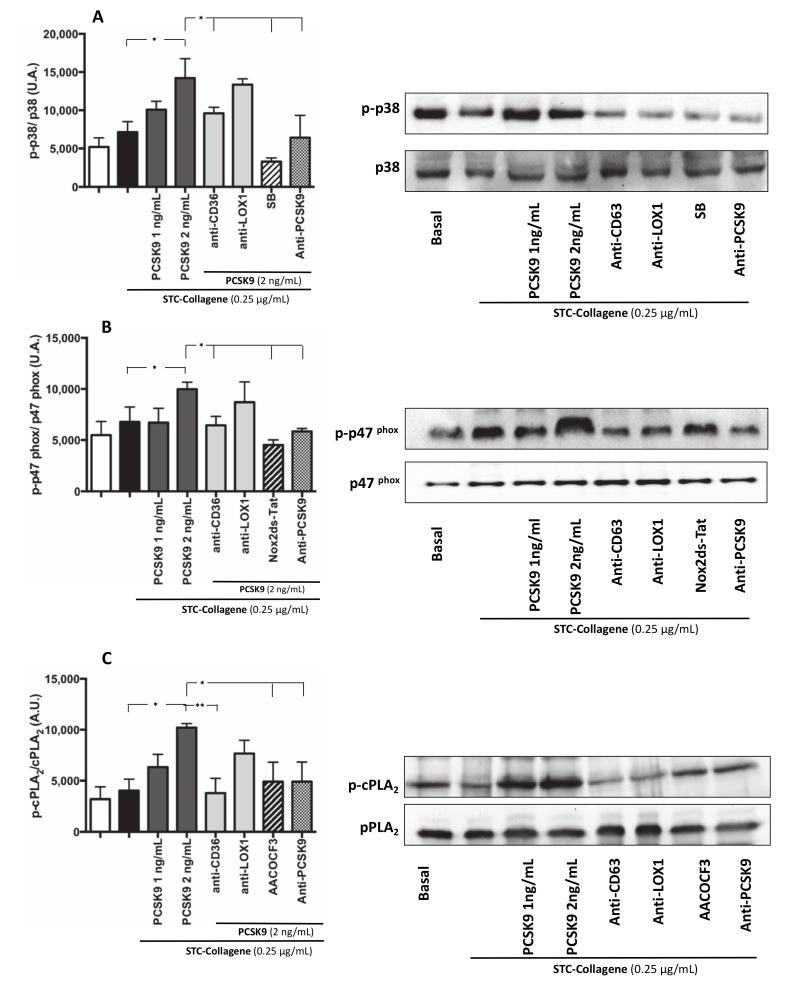
Intra-signalling pathway of platelet activation PCSK9-mediated: p38MAP Kinase, p47^phox^ and PLA_2_ phosphorylation. p38 phosphorylation, p47^phox^ phosphorylation and cPLA2 phosphorylation was analysed in platelets incubated with PCSK9 (1–2 ng/mL) and stimulated with subthreshold concentration (STC) of collagen (0.25 µg/mL) (**A–C**) in presence or less of anti-CD36, anti-LOX1, anti-PCSK9 (**A–C**) and incubated with or without SB (**A**), Nox2ds-tat (**B**) and AACOCF3 (**C**) (*n* = 3 independent experiments; *p < 0.05 **p < 0.001 in quantitative analysis). A representative Western blot of p38, p47phox and cPLA2 phosphorylation (**B–C**).

To analyse the pathway involved in PCSK9-dependent platelet activation by ROS, we studied p47^phox^ translocation on the platelet membrane, the role of p38MAP kinase, which is implicated in p47^phox^ activation [16] as final and key event upstream of Nox2 activation, and cPLA_2_ phosphorylation, a key enzyme for generation of eicosanoids. STC collagen-stimulated platelets in the presence of PCSK9 (2 ng/mL) showed a significant increase in p38MAP kinase, p47^phox^ and cPLA_2_ phosphorylation compared to STC collagen platelets alone (Figure 5A–C); this effect was inhibited in platelets pre-incubated with anti-CD36, anti-PCSK9, p38 inhibitor, Nox2ds-tat and AACOCF3 (Figure 5A–C).

#### 3.2.4. PCSK9 and LDL-Mediated Platelet Activation

**Figure 6:**
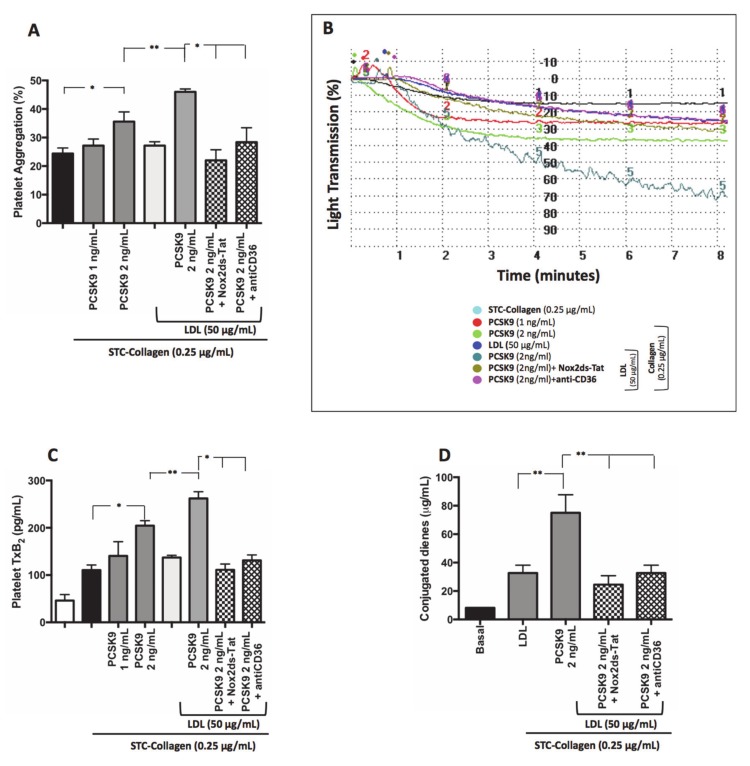
PCSK9 and platelet activation LDL-mediated. Platelet aggregation (**A**) with relative representative tracing (**B**), TxB_2_ production (**C**) and conjugated dienes formation (**D**) evaluated in washed platelet treated with PCSK9 alone (1–2 ng/mL) and stimulated with subthreshold concentration (STC) of collagen (0.25 µg/mL) in presence or less of exogenous LDL (50 µg/mL), Nox2ds-tat and anti-CD36. (*n* = 5; *p < 0.05, **p < 0.001 for paired analyses).

No significant changes in platelet activation parameters were detected in STC collagen-stimulated washed platelets in the presence of PCSK9 (1 ng/mL) compared to STC collagen-stimulated platelets alone (Figure 6A–C). Conversely, STC collagen-stimulated washed platelets in the presence of PCSK9 (2 ng/mL) showed an increase of platelet aggregation and TxB_2_ formation compared to STC collagen-stimulated platelets alone (Figure 6A–C). Of note, when PCSK9-treated platelets were also added LDL (50 µg/mL) a further increase in PA was detected, an effect blunted by CD36 or Nox2 inhibitors (Figure 6A–C). Finally, a significant increase in conjugated dienes formation was detected in STC collagen-stimulated washed platelets in the presence of PCSK9 (2 ng/mL) and LDL compared to STC collagen-stimulated platelets incubated with LDL alone (Figure 6D); PCSK9-induced formation of conjugated dienes was inhibited by CD36 or Nox2 inhibitors.

#### 3.2.5. PCSK9 Activates Platelets by CD36 Signalling

**Figure 7:**
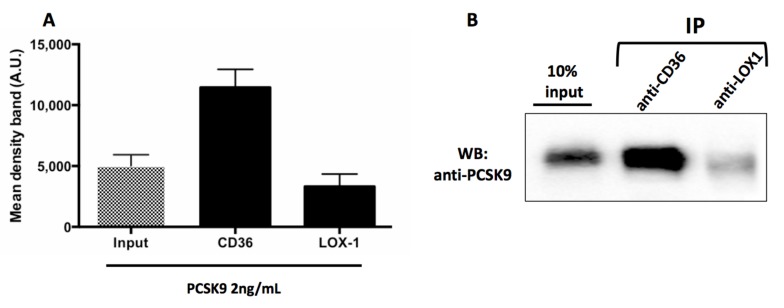
PCSK9 activates platelets by CD36 signalling. Densitometric analysis of Western blotting (WB) showing coimmunoprecipitation of CD36 and LOX1 with PCSK9 in human platelets stimulated with PCSK9 2 ng/mL (**A**). A representative Western blot bands of immune complexes CD36-PCSK9 and LOX1-PCSK9, the lane with 10% of the extract loaded before of IP (input) are shown (**B**). Results are representative of three independent experiments.

We investigated if PCSK9 directly interacts with CD36 and LOX-1, two major receptors for ox-LDL on platelets surface [17,18], by assessing a co-immunoprecipitation between PCSK9 and CD36 or LOX1. As reported in Figure 7, we demonstrated that PCSK9 co-immunoprecipitated with CD36 but not with LOX1. (Figure 7A,B).

## 4. Discussion

This study provides evidence that high circulating levels of PCSK9 are associated with increased platelet activation with a mechanism involving CD36 and eventually Nox2 activation. Such interaction is amplified in the presence of LDL. A direct link between PCSK9 levels and markers of platelet activity such as urinary TxB_2_ was observed in patients affected by AF [4] but the role of platelets was unclear also because it is still undefined as to whether platelet activation contributes to urinary TxB_2_ [19]. Hence, a definite association between PCSK9 levels and platelet reactivity is still lacking.

In our study, we analysed platelet function and ROS profiles comparing AF patients according to median PCSK9, i.e., 1.2 ng/mL, which is able to discriminate patients at an increased risk of CVEs [4]. We found that the population with PCSK9 levels > 1.2 ng/mL presented a significantly higher rate of platelet aggregation and recruitment coincidentally with higher levels of serum TxB_2_ formation, P-selectin release, ROS and ox-LDL. This finding was corroborated by in vitro study showing that 2 ng/mL PSCK9 was able to amplify the platelet response to collagen.

To investigate the underlying mechanism, we focused on down-stream pathways involving ROS-derived NADPH oxidase as this enzymatic pathway is activated by PCSK9 mediated increased cell activity in other models such as endothelial and smooth muscle cells [2].

A novelty of the present study is the demonstration that PA amplification by PCSK9 was associated with Nox2 activation suggesting a ROS-mediated pathway. Accordingly, PCSK9-mediated PA was associated with both p47phox and cPLA_2_ phosphorylation, which explains on one hand 8-iso-PGF2α over-production and on the other hand the enhanced TxA_2_ biosynthesis.

Recent data showed that in patients with familiar hypercholesterolemia anti-PCSK9 mAb treatment inhibits platelet reactivity but it is unclear if this effect is mediated by a direct interaction between PCSK9 and platelets or by LDL lowering [6]. Our data suggest that both contribute to platelet activation as in washed platelets PCSK9 per se behaves as a platelet agonist, an effect, however, that is amplified if PCSK9 is co-incubated with LDL. Such amplification by LDL was dependent on an enhanced formation of ox-LDL, a phenomenon already shown to amplify the platelet response to other agonists [15].

Finally, we demonstrated, by a co-immunoprecipitation method, that PCSK9 binds CD36 receptor so indicating that it activates aggregation by directly binding platelets. The down-stream signalling elicits platelet aggregation including ROS-derived Nox2 activation [9]. Taken together these data lead us, therefore, to hypothesize that the inhibition of platelet activation by PCSK9 mAb may occur via at least two mechanisms, one involving down-regulation of PCSK9 and the other LDL lowering.

This study has limitations and implications. The current data should be considered with caution as data were derived from an observational study and should be confirmed by an interventional study with a PCSK9 inhibitor drug. Moreover the direct interaction between PCSK9 and the platelet CD36 receptor should be better investigated by confocal immunofuorescence microscopy as reported by Demers and collaborators [20].

The fact that PCSK9 behaves as platelet agonist suggests that the beneficial effects of PCSK9 mAb may be dependent not only on LDL lowering but also by the inhibition of platelet function. This hypothesis, however, needs to be validated by an appropriate interventional study.

## 5. Conclusions

In conclusion, we demonstrated for the first time that PCSK9, at a concentration commonly found in the human circulation of AF patients, directly induces CD36-mediated platelet activation by amplifying Nox2 activity: this effect is amplified in the presence of LDL (Graphical Abstract).

## Figures and Tables

**Table 1:** Characteristics of study population.

	Total (*n* = 88)	Below Median PCSK9 (*n* = 44)	Above Median PCSK9 (*n* = 44)	*p*-Value
Age (years)	73.6 ± 7.7	74.1 ± 8.9	72.8 ± 6.9	0.424
Female sex (%)	48.9	43.2	54.5	0.394
Arterial hypertension (%)	88.6	93.2	84.1	0.314
Diabetes mellitus (%)	18.2	15.9	20.5	0.783
Heart failure (%)	14.8	15.9	13.6	0.764
Prior cerebrovascular events (%)	12.6	15.9	9.3	0.521
Prior cardiac events (%)	22.7	25.0	20.5	0.800
Antiplatelet therapy (%)	00.0			
Statins (%)	42.0	47.7	36.4	0.388
Proton pomp inhibitors (%)	43.0	38.6	47.6	0.514
CHA_2_DS_2_-VASc score	3.46 ± 1.42	3.64 ± 1.50	3.34 ± 1.40	0.341

**Table 2:** Correlation between markers of platelet activation and oxidative stress and PCSK9 levels.

	PCSK9	
	Rs	*p*-value
Platelet Aggregation	0.309	<0.001
Platelet Recruitment	0.485	<0.001
sP-selectin	0.330	<0.001
Serum TxB_2_ production	0.538	<0.001
Serum sNox2-dp	0.314	<0.001
Hydrogen peroxide	0.185	<0.001
Urinary 8-iso-PGE_2α_	0.336	<0.001
Ox-LDL production	0.550	<0.001

## Supplementary Materials

The following are available online at ’https://www.mdpi.com/2076-3921/9/4/296/s1, Figure S1: PCSK9 and platelet activation. Platelet aggregation (A) with relative representative tracing (B) and platelet TxB2 (C) evaluated in platelet rich plasma (PRP) treated with anti-CD36, anti-LOX1, Nox2ds-tat and anti-PCSK9 in absence ok PCSK9 and stimulated with STC of collagen (0.25 μg/mL) (n = 5) (*p* = ns for paired analyses).
