# Supplementary material for: PCSK9 Regulates Nox2-Mediated Platelet Activation via CD36 Receptor in Patients with Atrial Fibrillation
Source: Antioxidants (Basel). 2020 Apr 2;9(4):296. doi: 10.3390/antiox9040296 (PMC7222182; doi:10.3390/antiox9040296)
Supplement: Supplementary file 1 [file antioxidants-09-00296-s001.pdf]

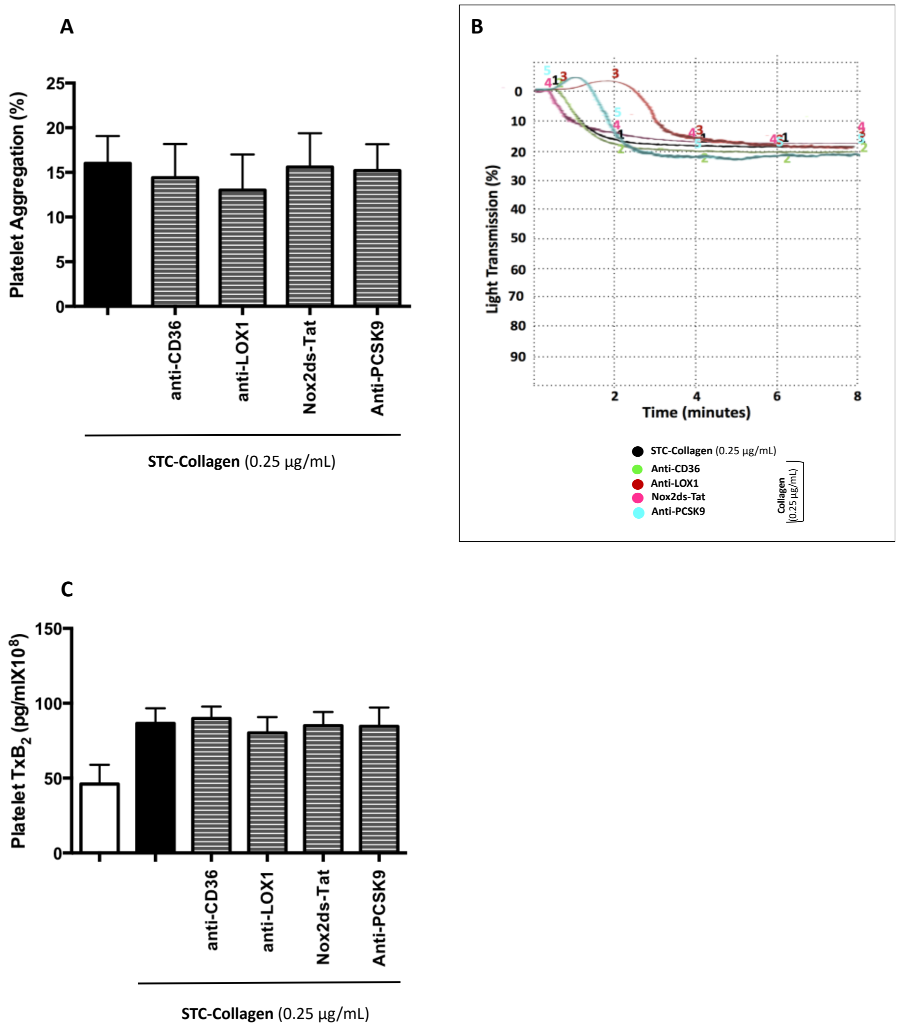

Figure S1. PCSK9 and platelet activation. Platelet aggregation (**A**) with relative representative tracing (**B**) and platelet TxB2 (**C**) evaluated in platelet rich plasma (PRP) treated with anti-CD36, anti-LOX1, Nox2ds-tat and anti-PCSK9 in absence ok PCSK9 and stimulated with STC of collagen (0.25 µg/mL) (n = 5) (p = ns for paired analyses).
